# SAMHD1 deficiency enhances macrophage-mediated clearance of *Salmonella* Typhimurium via NF-κB activation in zebrafish

**DOI:** 10.3389/fimmu.2025.1509725

**Published:** 2025-04-25

**Authors:** Alicia Martínez-López, Sylwia D. Tyrkalska, Francisco J. Martínez-Morcillo, Constantino Abenza-Olmos, Juan M. Lozano-Gil, Sergio Candel, Victoriano Mulero, Diana García-Moreno

**Affiliations:** ^1^ Immunology, Microbiology and Infectious Diseases, Instituto Murciano de Investigación Biosanitaria (IMIB)-Pascual Parrilla, Murcia, Spain; ^2^ Centro de Investigación Biomédica en Red de Enfermedades Raras (CIBERER), Instituto de Salud Carlos III, Madrid, Spain; ^3^ Departamento de Biología Celular e Histología, Facultad de BiologíaUniversidad de Murcia, Murcia, Spain

**Keywords:** SAMHD1, *Salmonella* typhimurium, NF-kB, zebrafish, innate immunity

## Abstract

**Introduction:**

Mutations in the gene encoding the protein containing the sterile alpha motif and the HD domain (SAMHD1) have been implicated in the occurrence of type I interferonopathies. SAMHD1 is also involved in blocking the replication of retroviruses and certain DNA viruses by reducing the intracellular amount of deoxynucleotide triphosphates (dNTPs). It has also been suggested that SAMHD1 negatively regulates interferon (IFN) and the inflammatory responses to viral infections; however, the functions and mechanisms of SAMHD1 in modulating innate immunity are still under study.

**Methods:**

In our laboratory, we have generated Samhd1-deficient zebrafish larvae using CRISPR-Cas9 and studied its role in the activation of nuclear factor kappa B (NF-κB) and the induction of type I IFN (IFN-I).

**Results:**

It was shown that Samhd1 deficiency results in the overactivation of the IFN-I response, assayed as the increased transcript levels of the Interferon Stimulated Genes (ISGs), but only if the larvae were stimulated with suboptimal doses of IFN-I. However, Samhd1-deficient larvae showed robust spontaneous activation of NF-κB, which led to increased larval resistance to *Salmonella enterica* serovar Typhimurium (STM) infection. Genetic experiments further showed that the activation of NF-κB in macrophages mediated the resistance of Samhd1-deficient larvae against STM.

**Discussion:**

These findings highlight the evolutionary conserved functions of SAMHD1 in the negative regulation of the inflammatory response of vertebrates and reveal, for the first time, a critical role for SAMHD1 in the regulation of NF-κB in macrophages to clear intracellular bacterial infection.

## Introduction

Homozygous mutations in the sterile alpha motif and HD domain-containing protein 1 (*SAMHD1*) gene are linked to Aicardi–Goutières syndrome (AGS), a rare inflammatory neurological disorder. This syndrome is characterized by the spontaneous production of type I interferon (IFN-I) and the upregulation of IFN-stimulated genes (ISGs), which resemble those of congenital viral infections (1–3).

SAMHD1 is a protein that plays a critical role in the immune response against viral infections (3–5), its deoxynucleoside triphosphate (dNTP) triphosphohydrolase (dNTPase) activity being one of its most studied antiviral characteristics. This activity plays a crucial role in regulating the DNA precursor pools in mammalian cells (6). In non-dividing cells of the myeloid lineage and in resting CD4-positive T cells, SAMHD1 restricts the infection of human immunodeficiency virus type 1 (HIV-1). However, in proliferating cells, SAMHD1 is phosphorylated by cyclin-dependent kinases (CDKs), which deactivates its restriction function and makes these cells susceptible to HIV-1 infection. Despite some controversy, SAMHD1 has also been proposed to exhibit nuclease activity on single-stranded RNAs, adding another layer to its antiviral features (7, 8). Beyond its direct antiviral activities, SAMHD1 has been suggested to play a role in the suppression of the innate immune responses to viral infections and inflammatory stimuli (5, 9, 10). This suppression is achieved through interactions with key proteins in the nuclear factor kappa B (NF-κB) and IFN-I pathways. Specifically, the SAMHD1 protein interacts with NF-κB1/2, reducing the phosphorylation of IκBα (NF-κB inhibitor alpha) and inhibiting the activation of NF-κB. SAMHD1 also reduces the induction of IFN-I by inhibiting the IKKϵ (inhibitor-κB kinase ϵ) phosphorylation of IRF7 (5, 11). Through these diverse mechanisms, SAMHD1 emerges as a crucial regulator of both antiviral defenses and inflammatory responses, underscoring its importance in maintaining immune homeostasis.

While SAMHD1 is associated with the immune response to viral infections, little is known about its possible role in immunity against bacterial pathogens. Similarly to viruses, bacteria have evolved to adapt to and counteract host inflammatory responses. *Salmonella enterica* serovar Typhimurium (STM) is a widely studied bacterial infection system due to the range of diseases caused. During infection, STM invades multiple cell types, including dendritic cells, epithelial cells, and macrophages. While macrophages typically serve as the frontline defense against invading bacterial pathogens, like the start of the restriction of STM infection (12, 13), they are essential for the establishment of systemic disease in a susceptible host (14–16). In macrophages, STM activates the canonical and non-canonical signaling pathways of NF-κB, which is the most important transcriptional regulator activating the inflammatory responses in the host against STM (17). In addition, one of the strategies that STM uses to evade the immune response and to favor its infection is the inhibition of the transduction pathways of NF-κB by the effector molecule SpvB from the pathogenicity island 2 type III secretion system (T3SS-2) (18).

To investigate the role of SAMHD1 during STM infection, we developed a Samhd1-deficient zebrafish model and examined the involvement of the NF-κB signaling pathway. The findings demonstrated that Samhd1 deficiency induced an increased IFN-I response only when larvae were exposed to a suboptimal dose of zebrafish IFN-I. Furthermore, a marked activation of NF-κB was observed, which was characterized by an increased NF-κB transcriptional activity. Notably, the enhanced NF-κB activation in Samhd1-deficient larvae correlated with increased resistance to STM infection, a response mediated by macrophages.

## Materials and methods

### Animals

Zebrafish (*Danio rerio* H.) were obtained from the Zebrafish International Resource Center and were mated, staged, raised, and processed as described (Westerfield, 2000 #23). The *Tg(NFkB-RE:eGFP)^sh235^* line, referred to as *nfkb:eGFP* (19) and *Tg(UAS:dn-nfkbiaa)^sd35^* (20), has been previously described. The experiments performed complied with the Guidelines of the European Union Council (Directive 2010/63/EU) and the Spanish RD 53/2013. The experiments and procedures were performed as approved by the Bioethical Committee of the University of Murcia (approval no. 669/2020).

### Salmonella Typhimurium infection assays

STM 12023 (wild type, WT) was used. Overnight cultures in Luria–Bertani (LB) broth were diluted 1:5 in LB with 0.3 M NaCl, incubated at 37°C until 1.5 optical density at 600 nm was reached, and finally diluted in sterile phosphate-buffered saline (PBS). Larvae at 2 days post-fertilization (dpf) were anesthetized in embryo medium with 0.16 mg/ml tricaine, and 10 bacteria per larvae were microinjected in the yolk sac. The larvae were allowed to recover in egg water at 28–29°C and were monitored for clinical signs of disease or mortality over 5 days.

### Analysis of gene expression

Total RNA was extracted from a pool of 25 zebrafish larvae (3 dpf) with the TRIzol reagent (Invitrogen, Carlsbad, CA, USA) following the manufacturer’s instructions and treated with DNase I, amplification grade (1 U/μg RNA; Invitrogen). The SuperScript VILO cDNA Synthesis Kit (Invitrogen) was used to synthesize first-strand cDNA with a random primer from 1 μg of the total RNA at 50°C for 50 min. Real-time PCR was performed with an ABI PRISM 7500 instrument (Applied Biosystems, Foster City, CA, USA) using SYBR Green PCR Core Reagents (Applied Biosystems). The reaction mixtures were incubated for 10 min at 95°C, followed by 40 cycles of 15 s at 95°C, 1 min at 60°C, and finally 15 s at 95°C, 1 min at 60°C, and 15 s at 95°C. For each mRNA, the gene expression was normalized to the ribosomal protein S11 gene (*rps11*) content in each sample using the Pfaffl method (27). The sequences of the used primers are listed in **Supplementary Table S1**. In all cases, each PCR was performed with triplicate samples and repeated at least twice with independent samples.

### In vivo imaging

To study the reporter activity of NF-κB, 2-dpf *nfkb:egfp* larvae were anesthetized in embryo medium with 0.16 mg/ml buffered tricaine. Images of complete larvae were taken using a Leica MZ16FA fluorescence stereomicroscope. The fluorescence intensity from three biological replicates, each containing several larvae, was obtained and analyzed with ImageJ (FIJI) software.

### CRISPR/Cas9, plasmid injections, and chemical treatments in zebrafish

The CRISPR RNA (crRNA) for zebrafish *samhd1* (**Supplementary Table S2**) and the negative control crRNA (catalog no. 1072544, crSTD), as well as the tracrRNA (*trans*-activating tracrRNA; catalog no. 1072533), purchased from Integrated DNA Technologies (IDT, Coralville, IA, USA), were resuspended in nuclease-free duplex buffer to 100 µM. Of each, 1 μl was mixed and incubated for 5 min at 95°C for duplexing. After removing from the heat and cooling to room temperature, 1.43 µl of the nuclease-free duplex buffer was added to the duplex [guide RNA (gRNA) and crRNA + tracrRNA], giving a final concentration of 1,000 ng/µl. The injection mix was then prepared by mixing 1 µl of the duplex, 2.55 µl of the nuclease-free duplex buffer, 0.25 µl of Cas9 nuclease V3 (IDT, catalog no. 1081058), and 0.25 µl of phenol red, giving final concentrations of the gRNA duplex (250 ng/µl) and of Cas9 (500 ng/µl). The prepared mix was microinjected into the yolk of one-cell-stage embryos (0.5–1 nl per embryo) using a microinjector (Narishige, Amityville, NY, USA). The same amounts of gRNA were used in all the experimental groups. The efficiency of the gRNA was checked by amplifying the target sequence with a specific pair of primers (**Supplementary Table S1**) and the TIDE webtool (https://tide.nki.nl/) (**Supplementary Figure S1**).

pcDNA-IFNphi3 (GenBank accession no. NM_001111083) (21) was microinjected into the yolk of one-cell-stage embryos (1 pg/embryo). The 1-dpf embryos were manually dechorionated and 24 h post-fertilization treated by chemical bath immersion at 28°C. Baricitinib (Bar; MedChemExpress, Monmouth Junction, NJ, USA) was added to the water at 10 µM and BAY11-7082 (MedChemExpress) at 10 nM and renewed every day for the duration of the experiment (6 days). Incubation was carried out in 10-ml plates containing ≈100 larvae/well in egg water (including 60 μg/ml sea salts in distilled water) supplemented with 0.1% dimethyl sulfoxide (DMSO).

### Statistical analyses

All statistical analyses were performed in GraphPad Prism 8. Data are shown as the mean ± SEM and were analyzed using one-way analysis of variance (ANOVA) and Tukey’s multiple range test to determine differences between groups. The differences between two samples were analyzed using Student’s *t*-test. A log-rank test was used to calculate the statistical differences in the survival of the different experimental groups.

## Results

### IFN stimulation is required to trigger an IFN-I response in Samhd1-deficient zebrafish larvae

**Figure 1:**
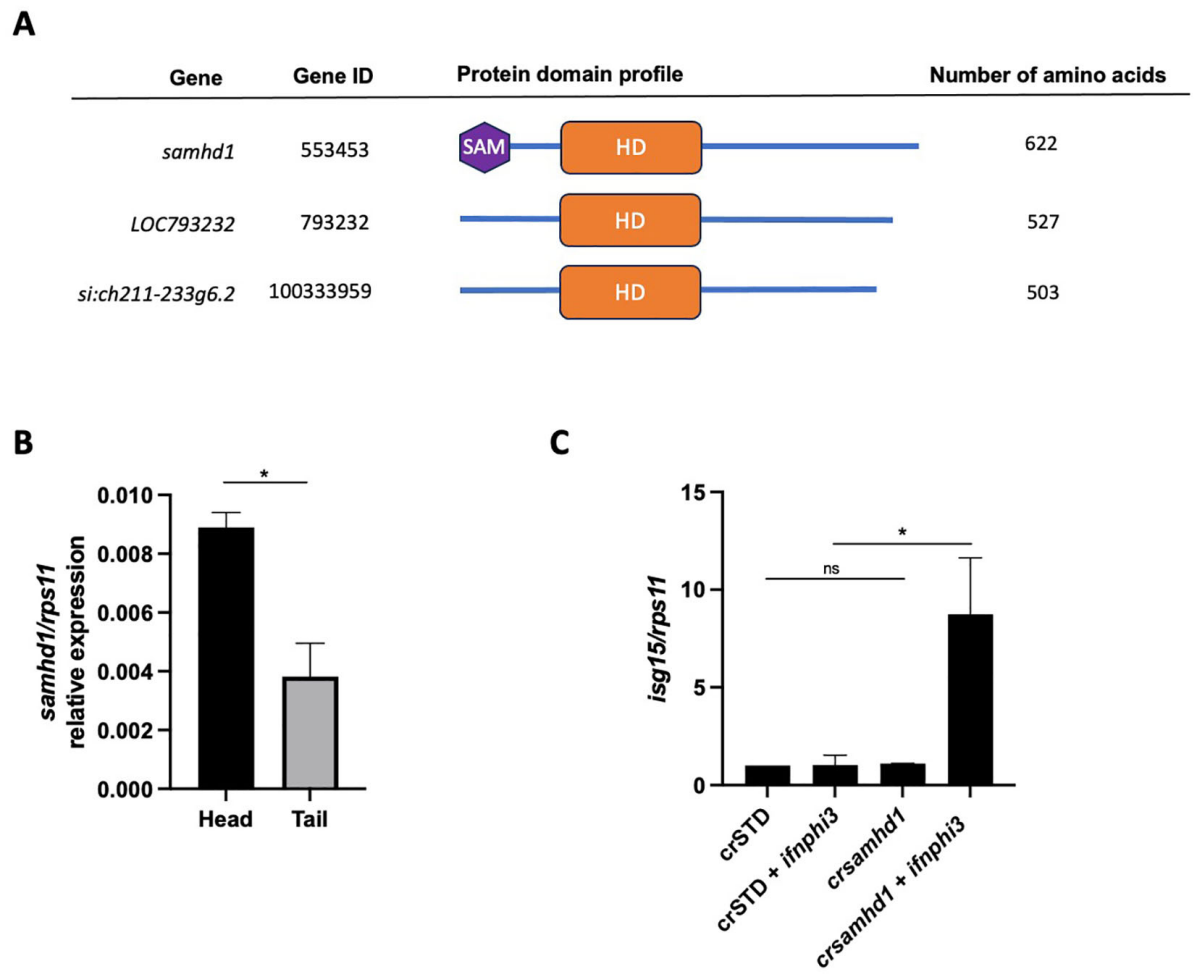
Interferon (IFN) stimulation is required to trigger interferon-stimulated gene (ISG) upregulation in Samhd1-deficient zebrafish larvae. **(A)** Diagram indicating the protein domains. **(B)** Transcript levels of *samhd1* by reverse transcription PCR (RT-PCR) in the head and tail of zebrafish larvae. The average from three independent experiments, each with 25 pooled heads or tails, is shown. **(C)** Transcript levels of *isg15* by reverse transcription quantitative PCR (RT-qPCR) in the head of zebrafish larvae injected with gRNA *(samhd1* or STD)/Cas9 complexes with or without 1 pg/egg of pcDNA-IFNphi3. *Bars* show the mean ± SEM of three independent experiments, each with 25 pooled heads. Data are represented as fold change from control (STD). *P*-values were calculated using Student’s *t*-test in **(B)** and using one-way ANOVA and Tukey’s multiple range test in **(C)**. *ns*, not significant. *p ≤ 0.05.

An annotated *samhd1* ortholog gene (Gene ID: 553453) to the human gene *SAMHD1* was found in chromosome 23 of the zebrafish genome (ENSDARG00000071288.4) (**Figure 1A**). In addition, two zebrafish *samhd1* paralogs were also annotated as *LOC793232* (Gene ID: 793232) and *si:ch211-233g6.2* (Gene ID: 100333959), both located in chromosome 1 (ENSDARG00000099421.2 and ENSDARG00000104626.2), which shared between them 94% of identity, while 58% and 57%, respectively, with the original *samhd1* gene. Importantly, the two *samhd1-*like sequences lacked the SAM domain (**Figure 1A**), which is critical for the regulation and activation of mammalian SAMHD1 (22), suggesting their specialization in other functions. Consequently, in this work, we only focused on the *samhd1* gene.

Similarly to Whiters et al. (23), we decided to measure the ISG expression in larval heads, given that the expression of *samhd1* was higher in heads than in tails (**Figure 1B**). We next inactivated *samhd1* using the CRISPR/Cas9 technology in one-cell-stage embryos (with a knockdown efficiency of 83.1%) (**Supplementary Figure S1**). We were not able to detect any significant difference in the expression levels of *isg15* between the Samhd1-deficient and the control zebrafish larvae (**Figure 1C**). However, the addition of a suboptimal dose of the zebrafish IFN-I *ifnphi3* was able to robustly increase the *isg15* transcript levels in Samhd1-deficient larvae, but not in their sibling controls (**Figure 1C**). Of note is that a suboptimal dose of 1 pg/egg of IFN-I *ifnphi3* was used in the experiment (**Supplementary Figure S2**).

### Samhd1-deficient zebrafish larvae show spontaneous activation of NF-κB and high resistance to Salmonella Typhimurium infection

**Figure 2:**
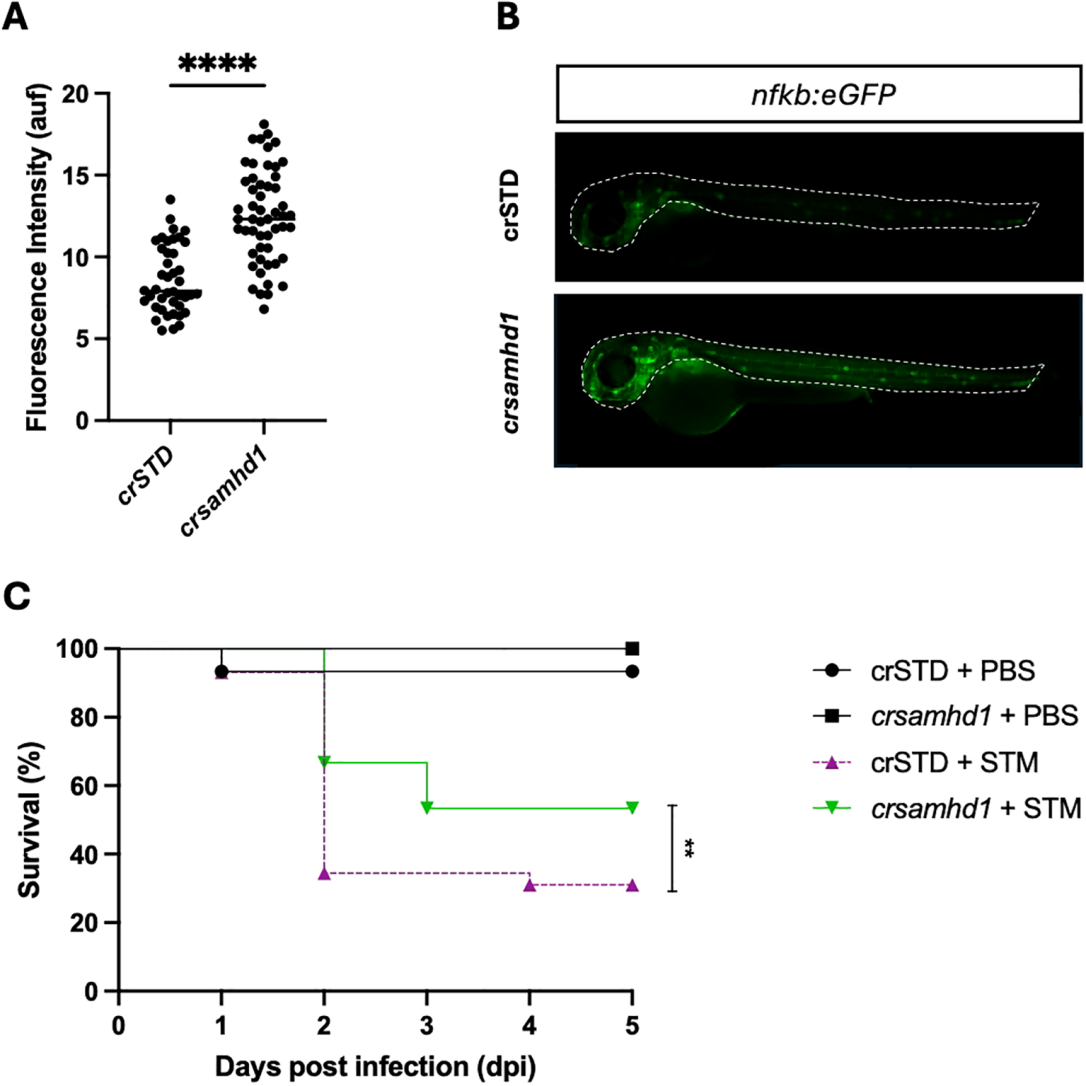
Samhd1 deficiency results in the spontaneous activation of nuclear factor kappa B (NF-κB) and hyperresistance to infection with *Salmonella enterica* serovar Typhimurium (STM). **(A)** Real-time visualization of NF-κB activation in Samhd1-deficient zebrafish larvae. The *nfkb:eGFP* reporter zebrafish line injected with gRNA (*samhd1* or STD)/Cas9 complexes were analyzed using fluorescence microscopy and quantified. *Each dot* represents a larva, and the mean ± SEM for each experimental group is also shown. **(B)** Representative images of whole larvae are shown, and the region of interest (ROI) used to quantify the fluorescence in the *nfkb:eGFP* reporter line is indicated as a *dot line* in the images. **(C)** Zebrafish one-cell embryos were injected with gRNA (*samhd1* or STD)/Cas9 complexes, dechorionated, and infected at 2 days post-fertilization via the yolk sac with STM at a multiplicity of infection (MOI) of 10 or phosphate-buffered saline (PBS), with the number of surviving larvae counted daily during the next 5 days. A total number of >100 specimens/treatment. *P*-values were calculated using Student’s *t* test in **(A)** and long-range test in **(C)**. **p ≤ 0.01, ****p ≤ 0.0001.

**Figure 3:**
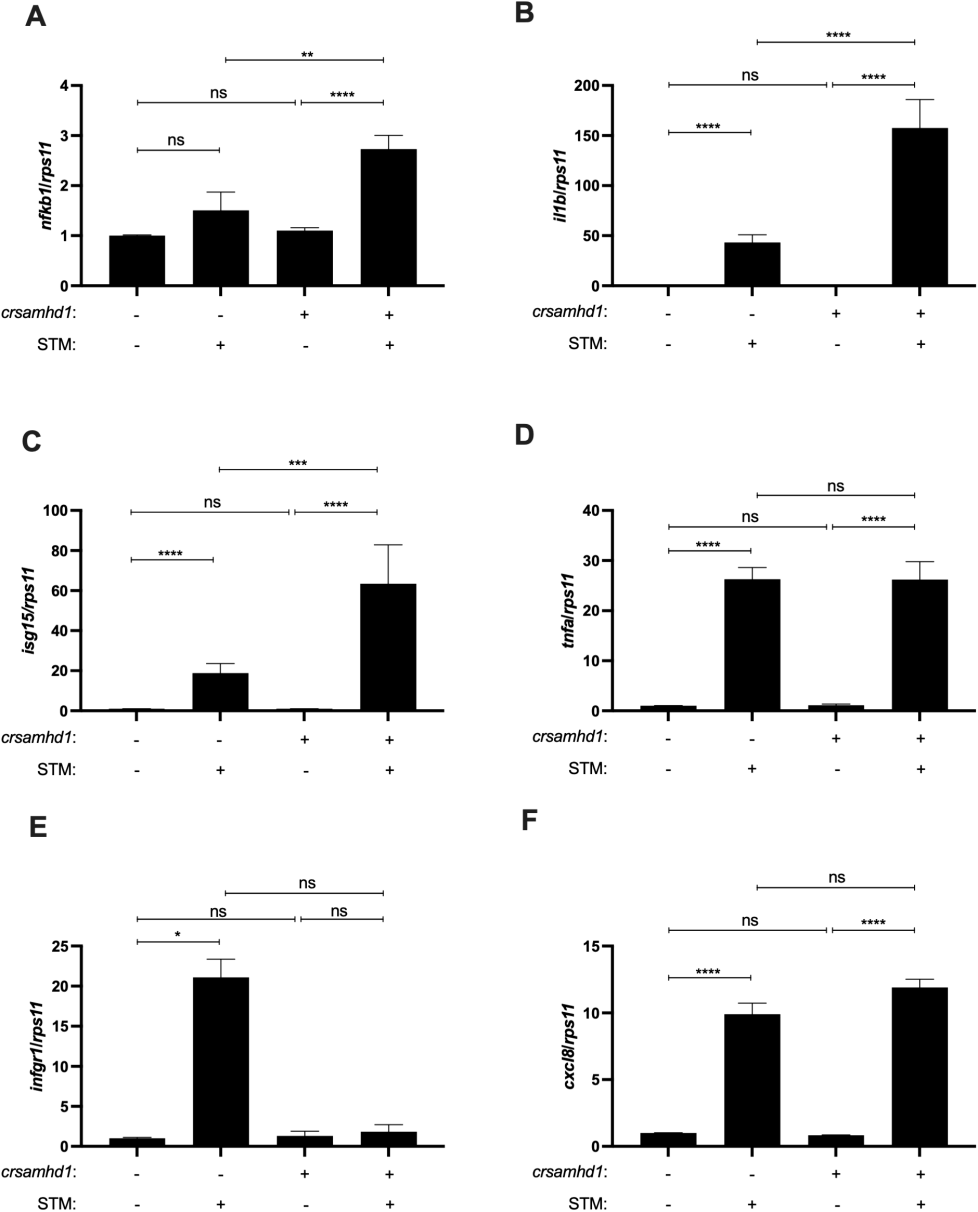
Samhd1 deficiency leads to an activated inflammatory state. **(A–F)** Transcript levels of *nfkb1* **(A)**, *il1b* **(B)** *, isg15* **(C)**, *tnfα* **(D)** *, ifng1r* **(E)**, and *cxcl8a* **(F)** in larvae injected with gRNA (*samhd1* or STD)/Cas9 complexes infected or not with *Salmonella enterica* serovar Typhimurium (STM) for 24 h assayed using reverse transcription quantitative PCR (RT-qPCR). *Bars* show the mean ± SEM of three independent experiments, each with 25 pooled larvae. Data are represented as fold change from control. *P*-values were calculated using one-way ANOVA. *ns*, not significant. *p ≤ 0.05, **p ≤ 0.01, ***p ≤ 0.001, ****p ≤ 0.0001.

To study the inflammatory process in Samhd1-deficient zebrafish larvae, the transcriptional activity of NF-κB was analyzed using the reporter line *nfkb:eGFP*. As seen in **Figures 2A, B**, Samhd1-deficient larvae showed stronger NF-κB reporter activity than control larvae. As NFKB protects murine models from STM infection and this pathogen is able to hijack NF-κB with its effector protein SpvB (18), we next studied whether Samhd1-deficient zebrafish larvae were more resistant to STM infection than their WT counterparts. The results confirmed this hypothesis, showing that Samhd1-deficient zebrafish larvae were more resistant to STM infection, with almost a 30% higher survival than WT larvae (**Figure 2C**). Moreover, the gene expression analysis after 24 h of infection showed that the transcript levels of *nfkb1* significantly increased in Samhd1-deficient infected larvae (**Figure 3A**) compared with control larvae. In addition, the expression of the *il1b* and *isg15* transcripts also increased in Samhd1-deficient larvae following infection (**Figures 3B, C**). Interestingly, the mRNA levels of *tnfα and ifng1r* from the Samhd1-deficient infected larvae did not overcome those of the control post-infection (**Figures 3D, E**). Finally, the transcript levels of *cxcl8a* were unaffected by Samhd1 deficiency independently of infection (**Figure 3F**). Collectively, these findings indicate that Samhd1-deficient larvae display a heightened inflammatory state, facilitating a more robust and accelerated response to pathogen infection compared with control larvae.

### Pharmacological inhibition of NF-κB abolishes the protective effect of Samhd1 deficiency in zebrafish larvae against Salmonella Typhimurium infection

**Figure 4:**
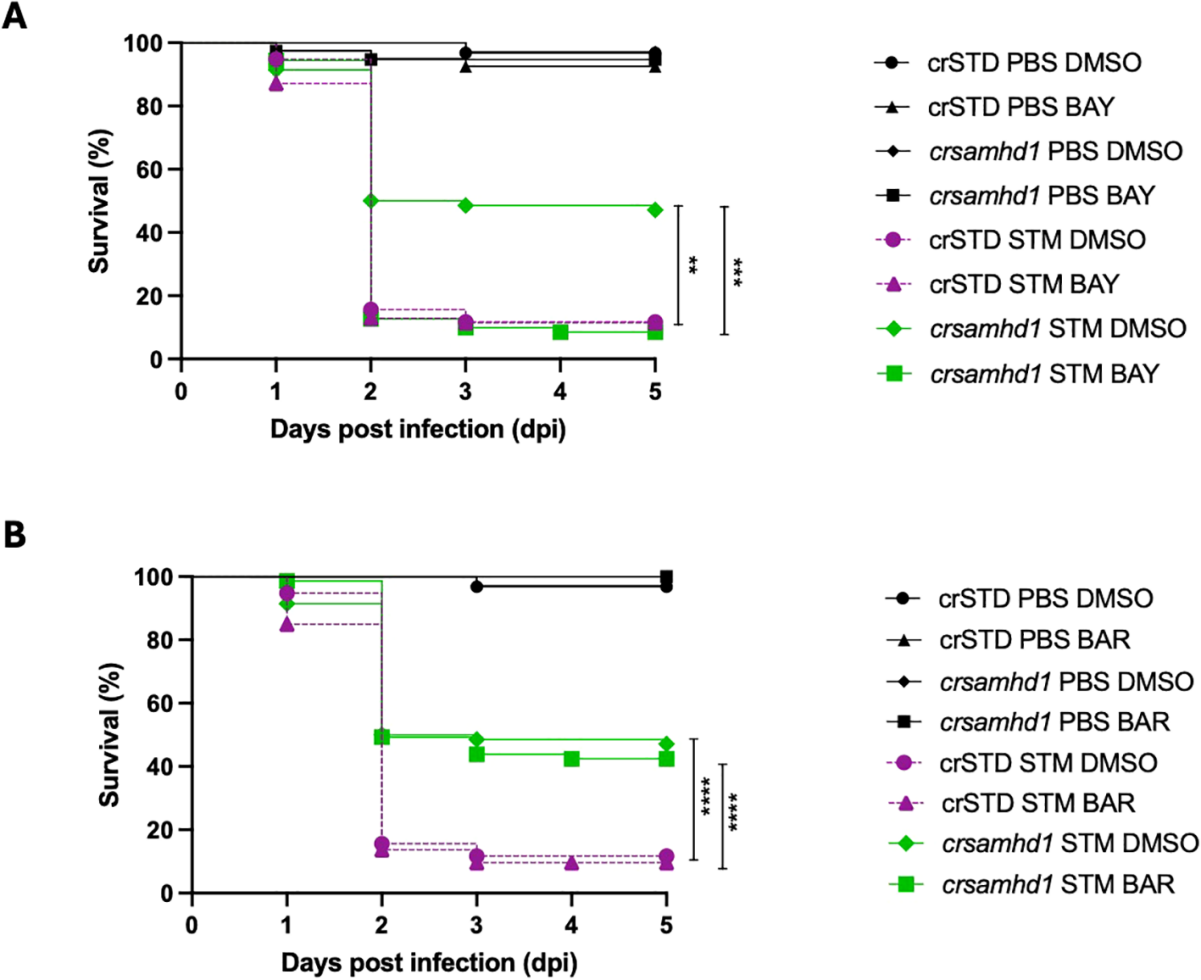
Pharmacological inhibition of nuclear factor kappa B (NF-κB) abolishes the protection of Samhd1-deficient zebrafish larvae to infection with *Salmonella enterica* serovar Typhimurium (STM). Zebrafish one-cell embryos were injected with gRNA (*samhd1* or crSTD)/Cas9 complexes, dechorionated at 24 h, and treated by bath immersion with 10 nM BAY11-7082 (BAY) **(A)** or with 10 μM baricitinib **(B)**. Drugs were renewed every day for the next 6 days. Controls were incubated with 0.1% dimethyl sulfoxide (DMSO). Larvae were infected at 2 days post-fertilization via the yolk sac with STM at a multiplicity of infection (MOI) of 10, with the number of surviving larvae counted daily during the next 5 days. A total number of >100 specimens/treatment. *P*-values were calculated using a long-range test. **p ≤ 0.01, ***p ≤ 0.001, ****p ≤ 0.0001.

To further validate the role of NF-κB in the resistance of Samhd1-deficient larvae to infection, we treated the larvae with BAY11-7082, an NF-κB inhibitor, and conducted a challenge with STM. As shown in **Figure 4A**, although the Samhd1-deficient larvae treated with the NF-κB inhibitor exhibited mortality rates comparable to those of WT larvae, the treatment fully abolished the increased bacterial resistance of Samdh1-deficient larvae (**Figure 4A**). Moreover, we used baricitinib, a JAK1/JAK2 inhibitor, which is able to suppress the IFN-I response in zebrafish larvae (24). Consistent with our expectations, treatment with baricitinib did not alter the susceptibility of Samhd1-deficient larvae to STM infection, which showed a survival rate approximately 30% higher than that of WT larvae and similar to that of untreated Samhd1-deficient larvae (**Figure 4B**). These findings support the hypothesis that the activation of NF-κB is crucial for the enhanced survival observed in Samhd1-deficient larvae.

### NF-κB activation in macrophages is responsible for the resistance of Samhd1-deficient zebrafish larvae to Salmonella Typhimurium infection

**Figure 5:**
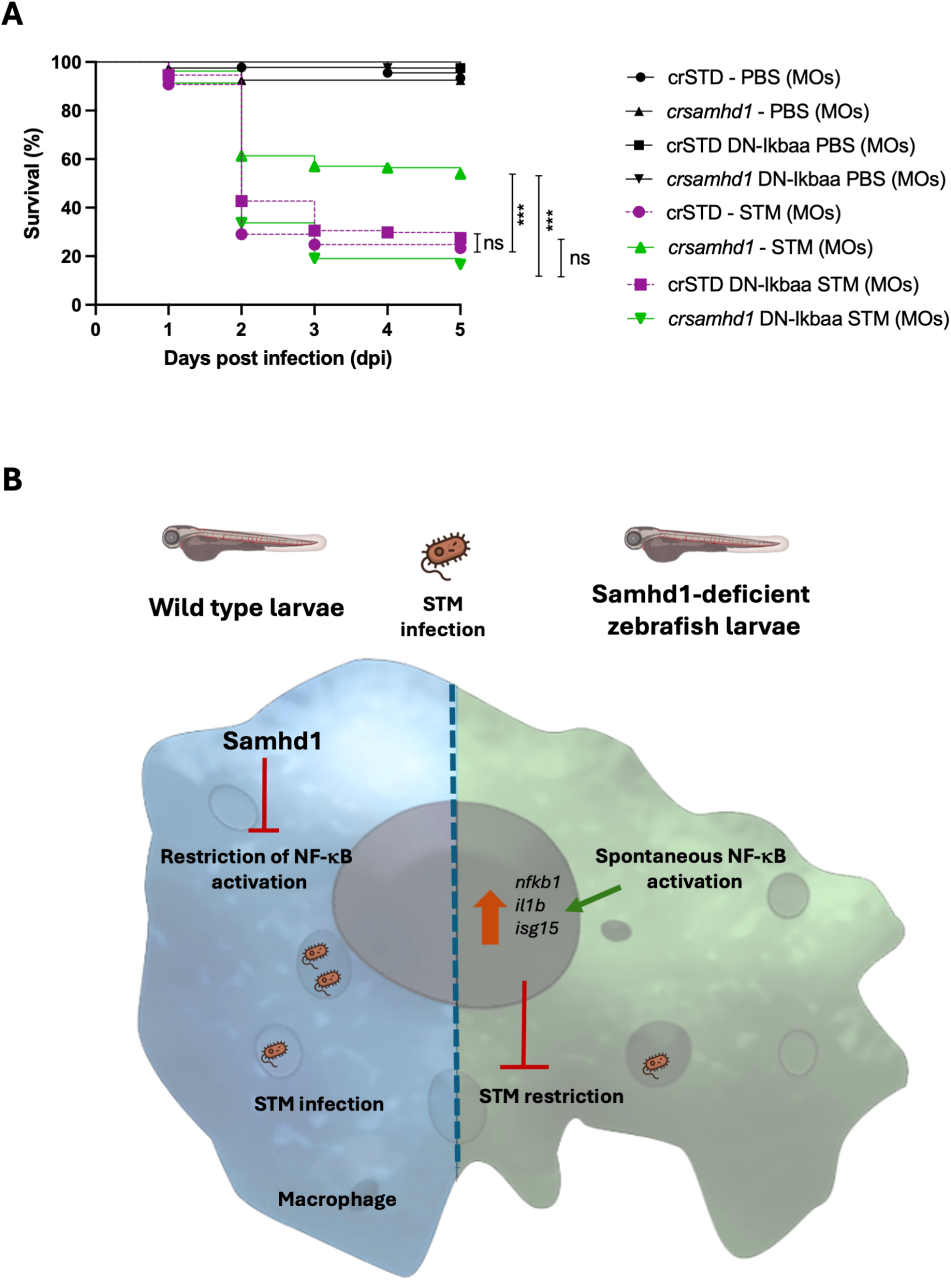
Genetic inhibition of nuclear factor kappa B (NF-κB) in macrophages abolishes the hyperresistance of Samhd1-deficient zebrafish larvae to infection with *Salmonella enterica* serovar Typhimurium. **(A)** The zebrafish line expressing a dominant negative (DN) form of Nfkbiaa, *Tg(UAS:dn-nfkbiaa)*, was outcrossed with the transgenic line *Tg(mpeg1:gal4)*, and one-cell-stage embryos were injected with gRNA (*samhd1* or STD)/Casp9 complexes and infected with STM as previously described. The average from two independent experiments with >80 larvae per group is shown. *P*-values were calculated using a long-range test. *ns*, not significant. ***p ≤ 0.001. **(B)** Model showing the restriction of NF-κB activation by Samhd1 in wild-type macrophages and the hyperresistance of Samhd-1deficient macrophages to STM infection through the induction of NF-κB activation.

The above results, together with the critical dual role of macrophages in STM dissemination and clearance (12–16), prompted us to examine whether the activation of NF-κB in the macrophages of Samhd1-deficient larvae mediated their hyperresistance to STM infection. To achieve this, we used the transgenic line *Tg(UAS:dn-nfkbiaa*), which expresses a dominant negative (DN) form of the nuclear factor of kappa light polypeptide gene enhancer in B-cell inhibitor, alpha a (*nfkbiaa*) and functions as an inhibitor of NF-κB (20). This line was outcrossed with the *Tg(mpeg1:gal4)* line, which allows exclusively expressing the DN-Nfkbiaa in macrophages. One-cell-stage embryos were injected with *samhd1* crRNA/tracrRNA/Cas9 complexes and subsequently infected with STM, as previously described. Larvae with macrophages expressing DN-Nfkbiaa lost their hyperresistance to infection (**Figure 5A**), behaving similarly to control larvae, indicating that the activation of NF-κB in Samhd1-deficient macrophages is essential for their enhanced resistance to STM infection. **Figure 5B** shows a schematic model of the restriction of NF-κB activation by Samhd1 in WT macrophages and the hyperresistance of Samhd-1-deficient macrophages to STM infection through the induction of NF-κB activation.

## Discussion

The role of SAMHD1 in modulating the immune response has been extensively studied in human and mouse models, where it has been shown to negatively regulate both the innate and adaptive immunity following viral infections or inflammatory stimuli, primarily through its interactions with proteins in the NF-κB and IFN-I pathways (4, 5, 11). As in other studies in zebrafish, in our model of Samhd1-deficient larvae, the upregulation of ISGs was moderated compared with that in human or mouse models and was only observed when a suboptimal stimulus was added (23). However, our zebrafish model, similar to the mouse model, did not develop any observable neurological phenotype (25, 26), contrary to what occurred in other zebrafish models (23, 27). The variability of the phenotypes found between studies could be due to the different techniques used to achieve the model, as transient gene knockdown by morpholinos (27) *versus* a stable mutant zebrafish line (23) or knockdown using CRISPR/Cas9 in this study. It is also important to consider that zebrafish live in a highly pathogen-enriched environment and that they possess compensatory mechanisms to avoid a permanent inflammatory state (28–31), and this could also be a reason for the type I interferonopathy models in zebrafish being very difficult to study and to determine a robust phenotype. Moreover, it is also logical to think that the knockdown *samhd1* model in this study does not show neurological phenotypes as it does not have a constitutive activation of IFN-I, which might be directly related to the overproduction of IFN-I (23, 27), as also mentioned in other AGS mouse studies (32, 33). These results also suggest that the characteristics of the animal facilities could also play a role in the study of the activation of the innate immune system in zebrafish, as the presence of microbial infections, particular microbiota, or other pro-inflammatory circumstances may contribute to AGS development.

One of the most interesting results in this study is the increased activation of NF-κB in Samhd1-deficient larvae. Similarly, this phenotype was also found after the depletion of *SAMHD1* in human and mouse models (4, 5, 25, 34, 35). This feature has been widely studied in viral infection systems to elucidate the restriction mechanism of SAMHD1, with different, even contradictory, results being obtained depending on the pathogen used. On the one hand, Kim et al. (4) observed that SAMHD1 deficiency activated the NF-κB innate immune pathway, resulting in increased viral replication through transcriptional activation of the human cytomegalovirus (HCMV) MIE gene promoter. On the other hand, Chen et al. (5) demonstrated that Sendai virus (SeV)-infected *SAMHD1*-silenced human monocytic cells or primary macrophages led to the increased nuclear accumulation of NF-κB, induction of IFN-I, and reduction of SeV nucleoprotein mRNA, which was attributed to the IFN-I-mediated inhibition of SeV replication.

All of these studies with SAMHD1 focused on the immune response to viral infections; however, little is known about its possible role in immunity against bacterial pathogens. In this context, we performed a challenge against STM in which the Samhd1-deficient larvae were more resistant to the infection. To further study the mechanism, we wanted to determine which cells were implicated in the activation of NF-κB and, hence, in the increased survival rate of Samhd1-deficient larvae. It is well known that *SAMHD1* is highly expressed in macrophages, dendritic cells, and CD4^+^ T cells, where it restricts retroviral infections (36–39). It has been recently reported that SAMHD1 disrupts the interaction between the upstream kinase TAK1 and IKKα or IKKβ, inhibiting the phosphorylation of IκBα and the activation of NF-κB (11). Furthermore, it is also well known that STM uses macrophages to replicate and establish systemic diseases (14–16, 40–43) and that its SpvB of the T3SS-2 has a potent and specific ability to prevent the activation of NF-κB by targeting IKKβ (18). Taking all these together, we decided to specifically block the activation of NF-κB in the macrophages of Samhd1-deficient larvae by expressing a DN-Nfkbiaa. The results demonstrated that the activation of NF-κB in macrophages was responsible for the high resistance of Samhd1-deficient larvae to STM infection.

In summary, this study is the first to reveal that Samhd1 plays a crucial role in restricting the macrophage-mediated clearance of STM infection. While previous studies have focused on the role of SAMHD1 in modulating the immune responses to viral infections, the findings of this study highlight its importance in bacterial immunity. It was demonstrated that Samhd1-deficient zebrafish larvae exhibit enhanced resistance to STM, which is driven by the increased activation of NF-κB in macrophages. This suggests that the loss of Samhd1 function allows for a more robust inflammatory response, facilitating a more effective pathogen clearance. These results open new avenues for exploring the role of SAMHD1 in bacterial infections and its potential as a therapeutic target in host–pathogen interactions.

## Data Availability

The raw data supporting the conclusions of this article will be made available by the authors, without undue reservation.

## Supplementary material

Supplementary Figure 1 Analysis of the efficiency of CRISPR/Cas9 edition. The edition efficiency of *gsamhd1*/Cas9 complexes was checked by amplifying the target sequence with a specific pair of primers (see **Supplementary Table S1**) and quantifying the rate of nonhomologous end joining mediated repair using the TIDE webtool (https://tide.nki.nl/). All insertions and deletions (INDELS) at the target site are shown.

Supplementary Figure 2 IFN stimulation. Transcript levels of *isg15* RT-qPCR in head of zebrafish larvae injected with *gRNAs (samhd1* or STD)/Cas9 complexes with or without 10 pg of pcDNA-IFNphi3 or 75 pg in wild type eggs **(A)**. Transcript levels of *isg15* RT-qPCR in head of zebrafish larvae injected with *gRNAs (samhd1* or STD)/Cas9 complexes with or without 1 pg of pcDNA-IFNphi3 **(B)**. Data is represented as fold change from control. *p*-values were calculated using Student’s *t*-test; ****p*≤ 0.001.
