# Supplementary figures and images for: SAMHD1 deficiency enhances macrophage-mediated clearance of Salmonella Typhimurium via NF-κB activation in zebrafish
Source: Front Immunol. 2025 Apr 25;16:1509725. doi: 10.3389/fimmu.2025.1509725 (PMC12062899; doi:10.3389/fimmu.2025.1509725)

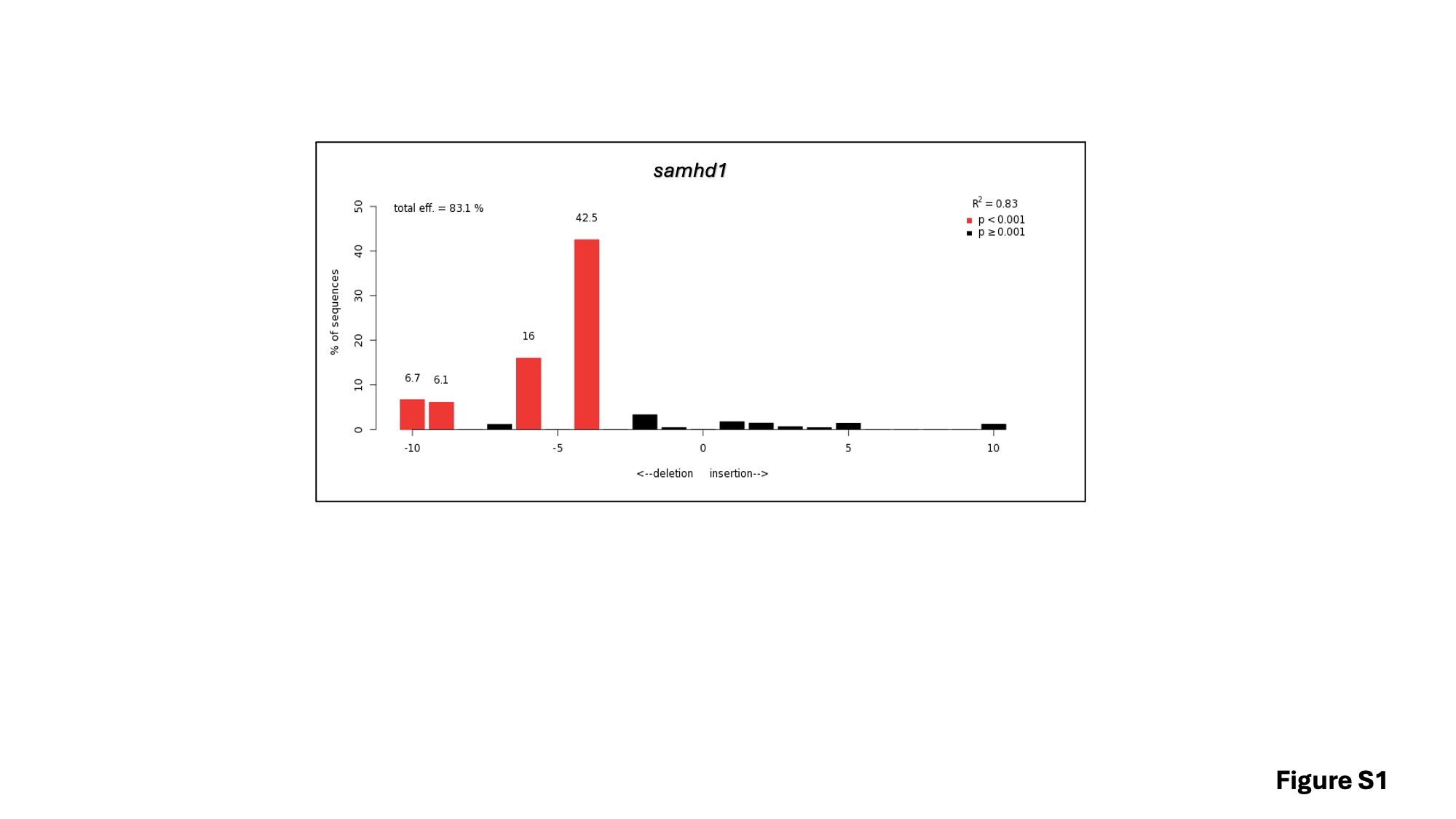

Supplement: Supplementary Figure 1 — Analysis of the efficiency of CRISPR/Cas9 edition. The edition efficiency of gsamhd1/Cas9 complexes was checked by amplifying the target sequence with a specific pair of primers (see Supplementary Table S1 ) and quantifying the rate of nonhomologous end joining mediated repair using the TIDE webtool (https://tide.nki.nl/). All insertions and deletions (INDELS) at the target site are shown. [file Image1.jpeg]

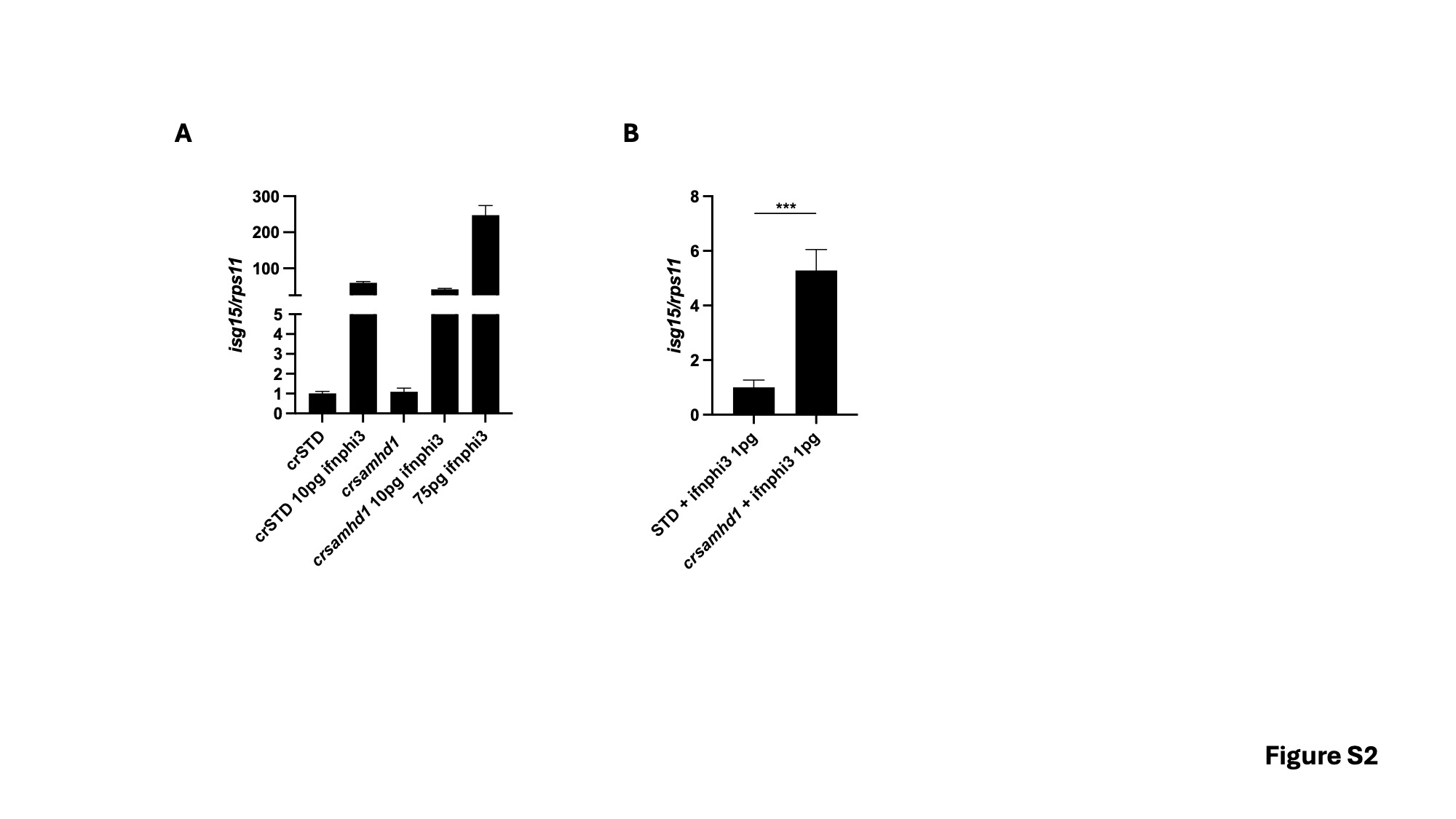

Supplement: Supplementary Figure 2 — IFN stimulation. Transcript levels of isg15 RT-qPCR in head of zebrafish larvae injected with gRNAs (samhd1 or STD)/Cas9 complexes with or without 10 pg of pcDNA-IFNphi3 or 75 pg in wild type eggs (A). Transcript levels of isg15 RT-qPCR in head of zebrafish larvae injected with gRNAs (samhd1 or STD)/Cas9 complexes with or without 1 pg of pcDNA-IFNphi3 (B). Data is represented as fold change from control. p-values were calculated using Student’s t-test; ***p≤ 0.001. [file Image2.jpeg]

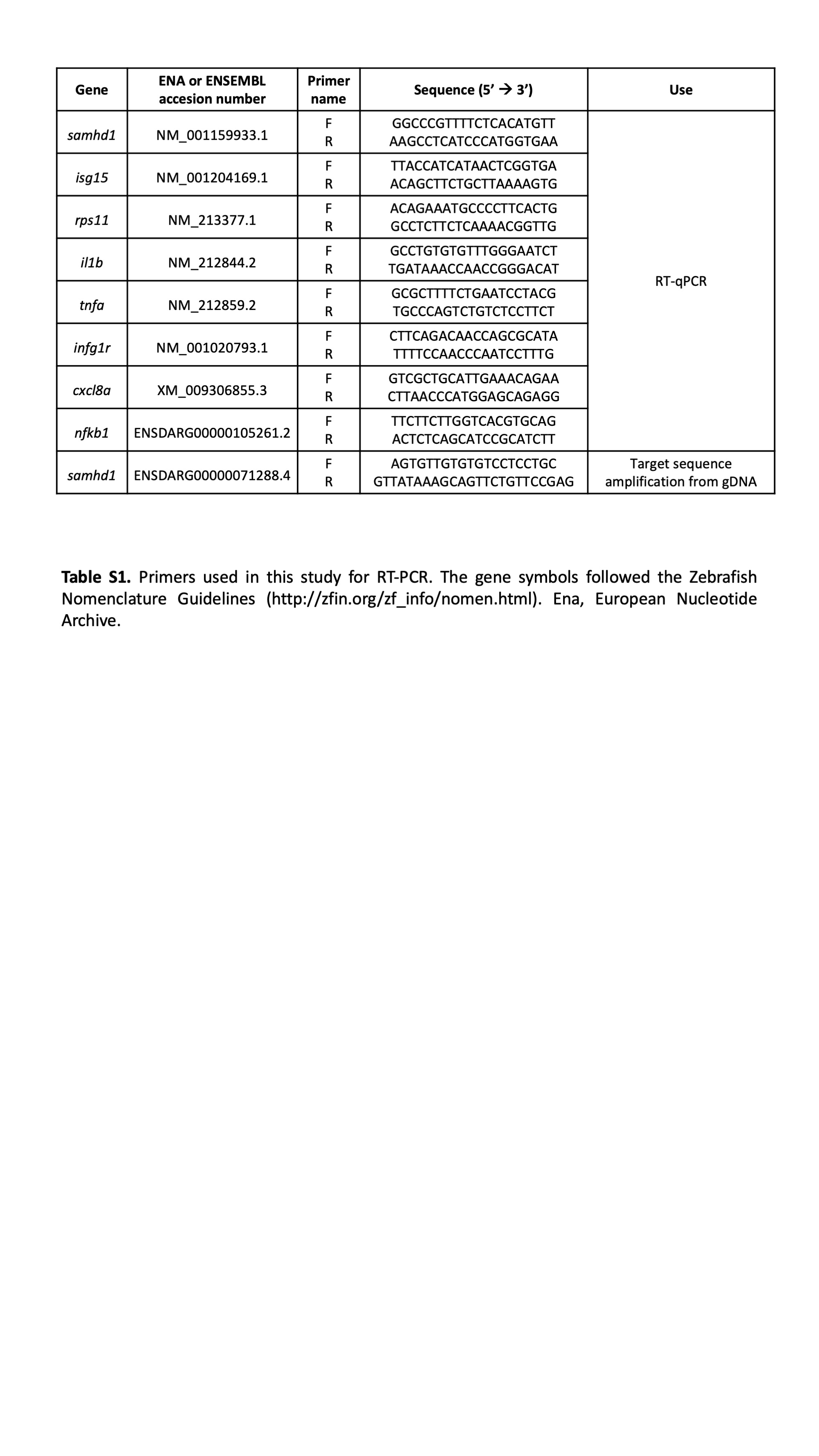

Supplement: Supplementary file 3 [file SupplementaryFile1.jpeg]

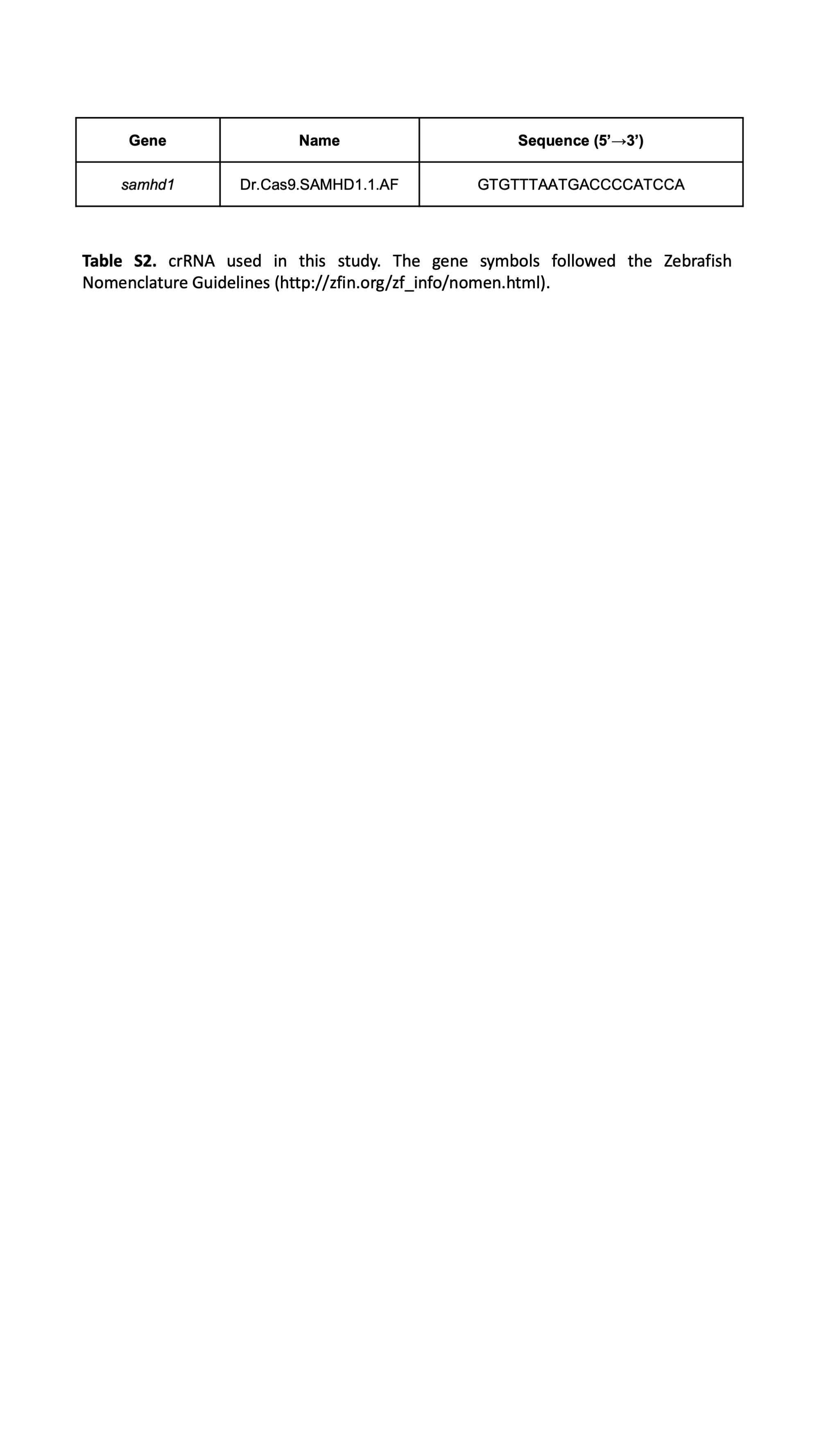

Supplement: Supplementary file 4 [file SupplementaryFile2.jpeg]
